# Long-Term Polyethylene (Bio)Degradation in Landfill: Environmental and Human Health Implications from Comprehensive Analysis

**DOI:** 10.3390/molecules29112499

**Published:** 2024-05-25

**Authors:** Vladyslav Redko, Lidia Wolska, Ewa Olkowska, Maciej Tankiewicz, Monika Cieszyńska-Semenowicz

**Affiliations:** Division of Environmental Toxicology, Faculty of Health Sciences with Institute of Maritime and Tropical Medicine, Medical University of Gdansk, Dębowa 23 A, 80-204 Gdansk, Poland

**Keywords:** polyethylene, long-term biodegradation, toxicological analysis, additives migration, environmental risk, health impact

## Abstract

This study investigates the process of long-term (bio)degradation of polyethylene (PE) in an old municipal waste landfill (MWL) and its implications for environmental and human health. Advanced techniques, such as ICP–ES/MS and IC–LC, were used to analyze heavy metals and anions/cations, demonstrating significant concentration deviations from control samples. The soil’s chemical composition revealed numerous hazardous organic compounds, further indicating the migration of additives from PE to the soil. Toxicological assessments, including Phytotoxkit F^TM^, Microtox^®^ bioassay, and Ostracodtoxkit^®^, demonstrated phytotoxicity, acute toxicity, and high mortality in living organisms (over 85% for *Heterocypris Incongruens*). An unusual concentration of contaminants in the MWL’s middle layers, linked to Poland’s economic changes during the 1980s and 1990s, suggests increased risks of pollutant migration, posing additional environmental and health threats. Moreover, the infiltration capability of microorganisms, including pathogens, into PE structures raises concerns about potential groundwater contamination through the landfill bottom. This research underscores the need for vigilant management and updated strategies to protect the environment and public health, particularly in older landfill sites.

## 1. Introduction

Polyethylene (PE) plastic stands out as one of the most widely utilized and versatile synthetic polymers in the world today [1]. Its history serves as a testament to human innovation, technological advancement, and the transformative impact of plastics on various industries. Low-density polyethylene (LDPE) finds applications in a diverse range of products, making it the most popular plastic choice among households [2,3]. Excessive production has led to a reduced usage lifespan for plastic products. The surplus amount of produced PE has consequently decreased the overall lifespan of plastic items.

Additional concern stems from the uncontrolled disposal of PE waste [4]. Among scientists, a discussion continues regarding the mechanisms of plastic degradation in the environment; initially, the focus was on the degradation within the polymer molecule structure, which could be demonstrated primarily using FTIR (Fourier transform infrared spectroscopy) technique. Numerous studies have analyzed the influence of factors such as UV, temperature, salinity, pH, etc., on various types of polymers [5,6,7]. Some biotechnologists have engaged in intensive research to seek/create modified bacteria capable of degrading polymer structures [8]. In recent years, there have been a few indications that the polymer degradation process is also linked to additives, which can be present in the polymer in amounts of up to 45–50% [9].

The primary mode of plastic degradation in environmental conditions involves the removal of additives from the plastic structure through a hydrolytic process [10]. This process leads to the extraction of additives—introduced to enhance physical and chemical properties—rendering the PE structure unstable. Progressive hydrolytic degradation results in the formation of cracks and delamination on the plastic structure, enabling environmental microorganisms to infiltrate, form colonies, create biofilms, and initiate biodegradation [11]. These processes are interconnected and are often synergistic in natural environments, collectively referred to as “(bio)degradation”.

During (bio)degradation, a range of organic and inorganic additives are released from the plastic structure into the surrounding environment, posing potential risks to ecosystems and human health. Organic contaminants include antimicrobials, antifogging additives, etc. [12]. Inorganic additives liberated from the PE structure encompass dyes and UV absorbers containing heavy metals, metal powders, flame retardants, etc. [12,13]. Moreover, heavy metals have a low capacity for migration in a soil environment, which means they can contaminate soil over an extended period [14]. The potential release of a wide array of substances presents challenges, as they can permeate groundwater from municipal waste landfills and contaminate soil, rendering it unsuitable for future use. This concern gains prominence as modern waste landfills, while equipped with safety measures, may not have been secured enough in the past when PE became widely used starting in the 1960s. Moreover, a lack of information was available about the complex impact of plastic wastes’ (bio)degradation in a soil environment, especially 60 years ago. In some studies [15,16,17], authors conducted investigations ranging from 2 months to ~30 years maximum, considering study of plastic waste degradation without a focus on its impact.

This extensive study encompasses a diverse range of analytical techniques aimed at investigating the cumulative impact of long-term (bio)degradation processes over an approx. 60-year span. Drawing upon insights gained from a realized preliminary investigation into the effects of 5-year polypropylene (PP) (bio)degradation, we have identified quite a lot of potential risks [18]. This initial study propelled us to extend an inquiry within the current research. A fortunate coincidence enabled us to gain access to an exceptionally valuable resource: the uncovered layer of a municipal waste landfill that had been in existence for 60 years. The investigation was concerned around two primary dimensions. The first facet scrutinizes the process of PE (bio)degradation within landfill environments. This study stream has already yielded substantial findings, including the identification of crude oil plastic materials, as documented in a previously published scientific work (Table S1). In the second dimension of the study, we delve deeply into the long-term repercussions of PE (bio)degradation, with a specific focus on its environmental ramifications. Drawing on insights from the preliminary analysis, we aim to elucidate the complex interplay between risk factors that affect both the environment and human health. The journey through this comprehensive exploration seeks to demystify the processes of (bio)degradation, evaluate their consequences, and contribute meaningfully to the expanding repository of knowledge that supports sustainable practices and informed decision-making.

Despite the growing prominence of technological recycling as a preferred method of waste management, juxtaposed against the deposition of plastic waste, humanity is confronted with the daunting challenge of managing vast quantities of already accumulated plastic waste. This challenge is further compounded by the (bio)degradation timeline, estimated at approximately 500 years, during which a broad spectrum of potentially toxic chemicals may be released. Moreover, the absence of comprehensive monitoring in Poland—contrasted with the periodic, extensive epidemiological investigations conducted in the UK around waste landfills—highlights a critical gap in the understanding and management of these issues. Given these considerations, it becomes evident that a significant and complex problem may arise in the future unless proactive measures are taken to address these challenges in the coming years.

## 2. Results

### 2.1. Sample Characterization

The soil samples obtained from the municipal waste landfill (MWL) were all associated with the (bio)degradation process due to their proximity to plastic waste (PW). Each sample correlated with a specific (bio)degradation period layer (Table 1). It is noteworthy that the plastic samples were analyzed in the initial stage of the investigation and all the collected PW were categorized as LDPE.

In soil characterization, the moisture content and pH values were determined for all the samples (Table 1). Compared to the control sample with a pH value of 7.24, all the MWL soil samples showed increased pH. The lowest pH (7.77) was recorded for soil from the MWL bottom at a level of −12 m, which yielded a pH of 7.75–7.77. The highest pH value was observed at a level of −1 m level (8.48) and at a level of −10 m (8.44). The moisture content, however, did not exhibit a clear trend or depth-related pattern. The control sample of the soil showed moisture contents ranging from 11.1% to 17.7%. The lowest values were found in soil from the MWL’s deepest levels (7.92% and 8.4%, respectively). The highest moisture content was recorded at −6 m (15.31%) and −10 m (14.9%).

### 2.2. Anionic and Cationic Composition of Soil

**Figure 1:**
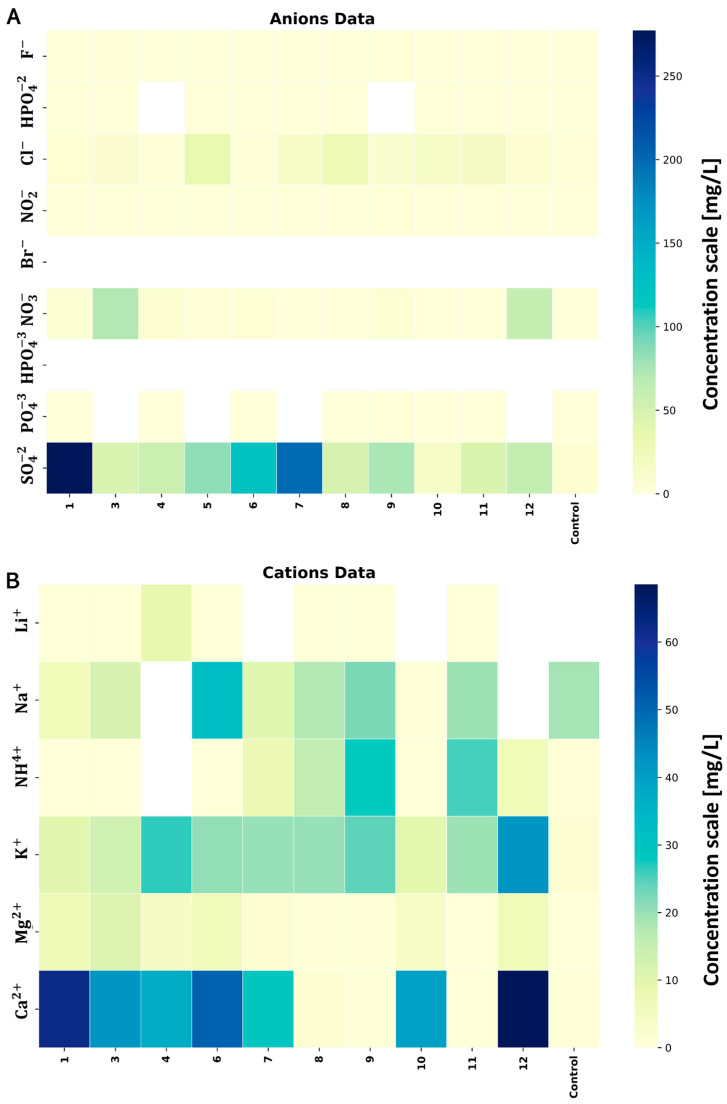
The heatmap represents the anionic (**A**) and cationic (**B**) concentrations (mg/g of dried soil) of soil samples tested and extracted using IC-LC. The analysis also includes control samples collected from a point not subjected to PE (bio)degradation.

In the subsequent investigation phase, disparities in ion composition between the soil samples from the MWL and the control sample were identified (Figure 1). The analysis of sample 2 was impossible to perform due to issues with the sample’s quality standards. Anions ($F^{-}$, $H{PO}_{4}^{-2}$, ${Cl}^{-}$, ${NO}_{2}^{-}$, ${Br}^{-}$, ${NO}_{3}^{-}$, ${SO}_{4}^{-2}$, $H{PO}_{4}^{-3}$, ${PO}_{4}^{-3}$) and cations (${Li}^{+}$, ${Na}^{+}$, ${NH}^{4+}$, $K^{+}$, ${Mg}^{2+}$, ${Ca}^{2+}$) were examined. Notably, a marked discrepancy in the total ion concentrations emerged between both sampling campaigns, comparing the cumulative concentrations of the MWL soil samples and the control sample distant from the MWL. While the total concentrations were elevated for anions, a reversible pattern was evident for cations. The highest cumulative concentrations were recorded for samples collected from the −6 m layer, measuring 1.43 mg/g, respectively.

Concerning the anions, ${SO}_{4}^{-2}$ exhibited a prominent presence across all the soil samples. The highest concentration was observed in samples from −0.3 m (2.77 mg/g) and −6 m (1.99 mg/g). The rest of the levels maintained a consistent concentration range of 0.45 mg/g to 0.49 mg/g, except for soil near the MWL bottom (0.15 mg/g). The control sample exhibited the lowest concentration (0.07 mg/g). A similar trend was observed for chlorides (Figure 1A), with the control sample showing a minor concentration of 0.02 mg/g. The ${Cl}^{-}$ concentrations increased with depth up to −6 m (−0.3 m: 0.05 mg/g; −1 m: 0.08 mg/g; −6 m: 0.35 mg/g). The lowest concentrations appeared in samples from −12 m (0.15 mg/g) and under the MWL (0.17 mg/g). ${NO}_{3}^{-}$ exhibited extremely high contrasts, with the first series showing concentrations of 0.72 mg/g (level −1 m) and 0.61 mg/g (level −6 m). ${Br}^{-}$ and ${SO}_{4}^{-2}$ were not detected in any samples.

Among the cations, the calcium concentration was noteworthy. The global trend was related to the cumulative concentrations (Figure 1B), which decreased with depth, and the bottom samples under the MWL demonstrated the lowest concentration compared to the control sample. The lowest concentration was exhibited at the −1 m (0.28 mg/g) layer, while the highest calcium concentration (0.64 mg/g) was at the −6 m level. The ammonium concentrations were exceptionally high in the soil sample collected from the bottom and under the MWL at −12 m (0.14 mg/g, 0.22 mg/g, and 0.21 mg/g—layer under MWL). A parallel situation emerged with the potassium concentrations in both collections.

### 2.3. Heavy Metals Analysis

**Figure 2:**
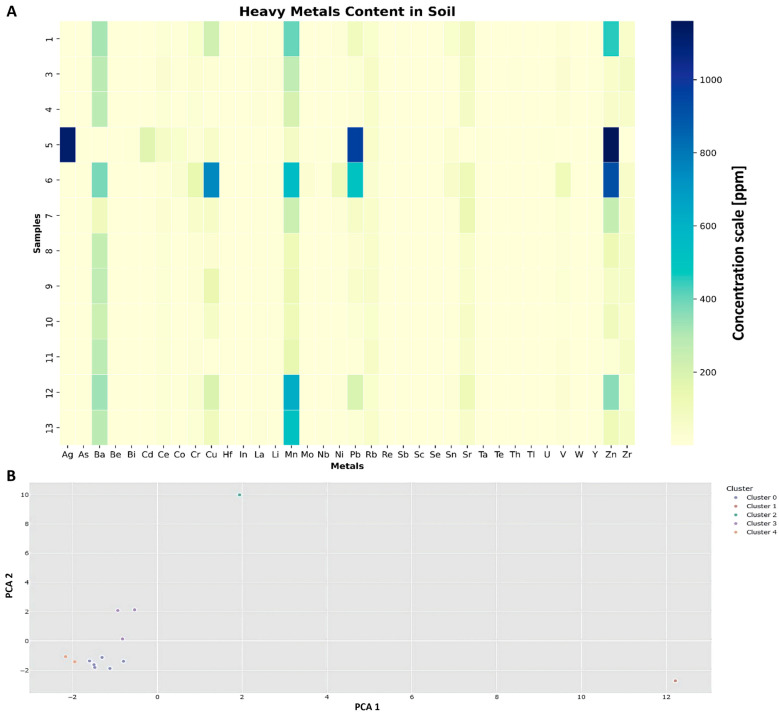
Results of heavy metal screening and clustering analysis in collected soil samples. (**A**) displays the outcomes of the heavy metal screening conducted on the collected soil samples. (**B**) illustrates the clustering analysis performed using machine learning techniques. The clustering was achieved through an unsupervised learning approach and aimed at solving the clustering problem based on their characteristics due to k-means algorithms.

The heavy metal content in the soil samples collected from the municipal waste landfill (MWL) is detailed in Figure 2. The concentrations of various heavy metals were meticulously analyzed and quantified; a machine learning technique was implemented to conduct data analysis with a clusterization analysis (k-means is good for data needing outliers’ consideration). The analysis of sample 2 was impossible to perform due to issues with the sample’s quality standards. The summary concentrations of these heavy metals (Figure 2A) in each soil sample provide an overview of the overall metal contamination within the landfill environment. Among the soil samples, the soil sample at −6 m exhibited the highest sum of metal concentrations, with a total of 3896.3 ppm, followed by the sample at the −12 m level (2104.8 ppm) and the control sample (1337.9 ppm).

A closer inspection of individual metal concentrations within each soil sample reveals distinct patterns (Figure 2A). For instance, the sample at −0.3 m demonstrated elevated levels of Cu (224.9 ppm) and Zn (460 ppm), whereas the sample from −6 m presented high concentrations of Cu (751.8 ppm) and Zn (918 ppm). The control sample displayed significant concentrations of Cu (39 ppm) and Zn (156 ppm), while the sample from −10 m displayed elevated Cu (67.6 ppm) and Zn (98 ppm) content.

The clustering analysis represented the same tendency (Figure 2B) and divided into five clusters. The results of the soil collected from the −6 m level were divided as separate clusters (cluster 1 and cluster 2); the total concentration of all the detected metals was the highest for these samples. Similarly, the results of the soil samples collected from the −10 m level were categorized in a separate cluster (cluster 3). Cluster 4 contained only two samples: samples from levels −1 m and −12 m (soil under MWL). However, it also included samples within the first sampling. The rest of the samples were dedicated to cluster 0.

The next step within the study encapsulates a meticulous examination of the concentrations of diverse bioavailable heavy metals within a spectrum of soil samples, each characterized by distinct pH values. The study presents a comprehensive picture of heavy metal behavior in response to varying pH conditions. The array of heavy metals investigated encompasses Ag, Al, As, Ba, Be, Bi, Ca, Cd, Ce, Co, Cr, Cu, Fe, In, K, La, Li, Mg, Mn, Mo, Na, Ni, P, Pb, S, Sb, Sc, Se, Sn, Sr, Te, Th, Ti, Tl, V, and Y. The pH values scrutinized in this analysis are pH 1, pH 2, and pH 5.3. A perusal of the dataset facilitates the discernment of salient trends regarding heavy metal concentrations in the context of differing pH environments.

#### 2.3.1. pH 1

Under the extremely acidic conditions of pH 1, the concentrations of various heavy metals manifest a general diminishment across the spectrum of the soil samples. Nevertheless, intriguing divergences in concentration levels arise, offering a glimpse into the intricate complexities of metal–soil interactions. Noteworthy revelations emerged for specific elements: Ag, Al, and As assert their presence with relatively heightened concentrations in select soil samples, most conspicuously observed in soil samples from −6 m and −10 m (both from first collection).

#### 2.3.2. pH 2

Transiting into the realm of moderately acidic conditions at pH 2, the concentrations of heavy metals exhibit discernible fluctuations. While the overall concentrations remain relatively subdued for most elements, certain metals, such as Cu, Fe, and Ni, exhibit modest elevations in specific soil samples, prominently seen at the −12 m level (including the soil sample under the MWL). This phase underscores the nuanced sensitivity of metal bioavailability to marginal shifts in pH.

#### 2.3.3. pH 5.3

The exploration culminates at pH 5.3, a near-neutral state reflective of many natural ecosystems. Here, a compelling trend unfolds, as several heavy metals exhibit heightened concentrations compared to their counterparts under lower pH conditions. Elements like Al, As, Ca, Cd, Cu, Fe, Mn, Ni, Pb, S, and Zn command attention with their enhanced presence within the extracts of the soil samples. This phenomenon underscores the pivotal role of pH as a regulator of heavy metal bioavailability. Notably, the transition towards neutral pH levels appears to augment the solubility and mobility of these metals, potentially making them more accessible for uptake by plants and other biota.

In synthesis, this extensive analysis underscores the intricate interplay between soil pH and heavy metal concentrations. Interestingly, these variations in heavy metal concentrations within different soil samples indicate complex interactions between the plastic waste and the soil matrix, which can be attributed to factors like waste composition, degradation processes, and soil properties.

### 2.4. Volatile and Semi-Volatile Organic Compounds

DCM (dichloromethane) extracts obtained from the control and MWL soil samples were analyzed using GC–MS/MS. The samples from levels −0.3 m and −6 m showed the highest total concentrations, which were 18 times higher compared to the control sample. In terms of the number of detected substances, the maximum was observed in soil samples from −1 m and −10 m (108 and 106 detected substances, respectively). Meanwhile, samples with the highest concentrations showed 87 (−0.3 m layer) and 68 (−6 m layer) substances. The lowest concentrations were detected below the −6 m layer, after which a significant decline was observed. The lowest total concentration was found in samples at the −12 m level (under the MWL), which was 36 times lower compared to the −6 m level. The results obtained from the collected samples underwent descriptive analysis, considering classes of substances and the presence/absence of halogenated compounds, which can appear in soil due to (bio)degradation processes. This comprehensive analysis of organic compound composition across multiple samples focuses on a wide range of compound categories, shedding light on the variations and trends observed within each category.

Alcohols: Alcohol compounds also exhibit substantial variation, with the samples from −6 m showing the highest concentration at 8 compounds, like the control sample. The samples from −12 m represented a medium number with 5 substances. Conversely, soil samples from the same level showcased the lowest count, each having only 1 compound.

Alkenes, aldehydes, and naphthalenes: While the soil from −12 m boasts the highest number of alkenes at 6 compounds, several samples, such as the soil samples from −6 m, −12 m (under MWL), and others, lack this specific compound type. Similarly, aldehydes are prominent in the soil from −0.3 m but are absent in multiple other samples. Naphthalenes are notably present in the soil from the −0.3 m level, yet they are absent in several other samples.

Esters and ketones: These compounds exhibit varying prevalence, with the samples from −6m having the highest number of esters (6), and the samples from −0.3 m containing the highest number of ketones (4). Multiple samples featured lower quantities of these compounds, suggesting diverse chemical profiles. The control sample represented the second-highest number of esters (5) and 0 ketones.

Halogenated substances: Among the levels, the sample from −12 m represented the highest number of halogenated substances. The control sample had only 1 substance. The −12 m level (under the MWL) represented 6 halogenated substances. The −10 m level showed a lack of such substances, including halogens.

Phthalates: Aromatic compounds exhibit notable diversity, with −12 m (under the MWL) having the highest count of 31 substances. Additionally, the presence of phthalate compounds is significant, particularly in the control sample and soil samples from −0.3 m and −6 m. Considering the total concentration of phthalates, the highest was detected at the −0.3 m level, and the −6 m level was the second-highest concentrated level. Additionally, levels below −6 m represented significantly lower concentrations. The control sample represented a concentration 105 times lower than the maximum. At the −12 m level (under the MWL), the total concentration was 5 times lower in contrast to the control samples.

### 2.5. Volatile Organic Compound Composition in Air

The VOCs’ air composition was evaluated and compared for all the collected air samples from different layers of the MWL. The study was conducted only once within the first sampling during the investigation. The root cause was the weather conditions. To conduct quality analysis, the internal standard (1-bromo-3-fluorobenzene) technique was applied. The highest total concentration of VOCs in the air was detected at the −10 m level (6465 µg/m^3^) contained under soil phase separated due to a metal barrier. The lowest total concentration was detected at the −0.3 m level (267.8 µg/m^3^). The level under the bottom of the MWL (−12 m) represented a total concentration equal to 835 µg/m^3^, which was 3 times higher than at the −0.3 m level. Within the investigation, an in-depth analysis of the chemical composition of air samples collected at various depths of the MWL was conducted as well, to identify trends and variations in the presence and abundance of the air content of each level.

The chemical analysis revealed significant differences in the chemical composition of the air samples at different depths (Table 1). At a depth of −0.3 m, the dominant class of organic compounds was aromatic hydrocarbons, with 6 substances. The second most prevalent class was aldehydes/ketones, consisting of 5 substances. Unique compounds like 1-hexanol and 1-octanol were observed only at this level. Moving to −1m depth, anhydrides/esters were the most abundant class, featuring 24 substances. Aromatic hydrocarbons ranked second with 12 substances. Notably, alcohols were absent at this level, and several substances from shallower levels were not detected. At −6m depth, hydrocarbons dominated the composition with 37 substances, while aromatic compounds came next with 25 substances. The absence of alcohols and ethers was a distinguishing feature at this level. The unique appearance of various hydrocarbons marked this depth. Descending to −10 m, hydrocarbons remained dominant with 38 substances, and aromatic compounds followed with 25 substances. Like the deeper levels, alcohols and ethers were not detected, and numerous hydrocarbons unique to this level were present. At the deepest level of −12 m, aromatic compounds, and hydrocarbons shared dominance with 10 substances each. Alcohols ranked second with 2 substances. Fewer substances were observed in other classes. Several unique compounds, both hydrocarbons and aromatic compounds, characterized this level. The abundance and presence of different classes of organic compounds showed notable fluctuations, likely driven by environmental factors.

### 2.6. Ecotoxicity Assessment

**Figure 3:**
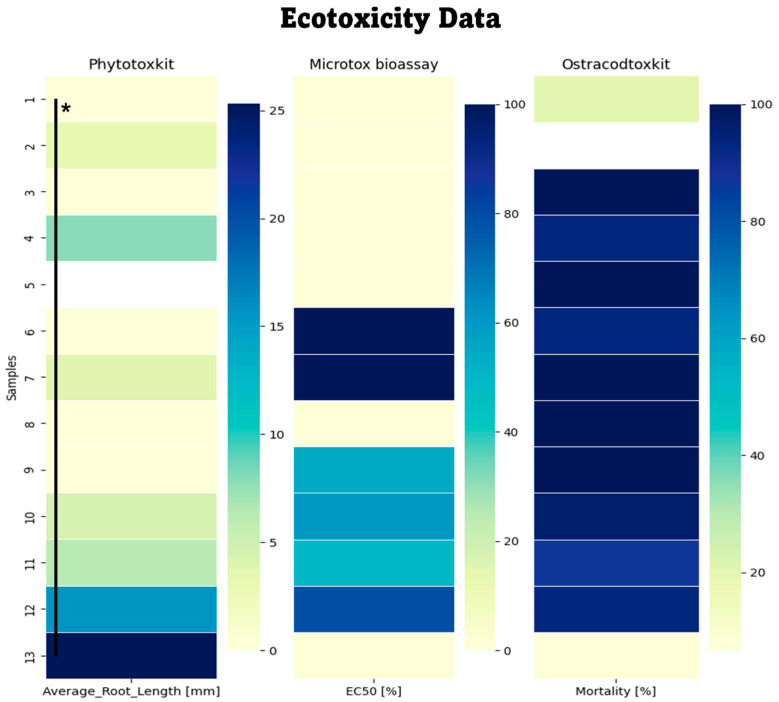
Ecotoxicity data were obtained from samples tested from the MWL and a control sample (n = 13). Average_Root_Length represents the results of the Phytotoxkit F^TM^, which assesses chronic toxicity. The * ANOVA test was performed with Python 3.11 (SciPy version 1.6.0). The statistical * stats for *S. sacharatum* are F-value = 89.63, *p*-value = 0.0043 (significantly different, *p* < 0.01). EC50 [%] represents the results of the Microtox^®^ bioassay test, which assesses acute toxicity. The Mortality [%] parameter represents the Ostracodtoxkit^®^, a “direct contact” ecotoxicity test sensitive to the potential influence of toxic substances in soil.

The ecotoxicity of the soil samples collected from the vicinity of the MWL, distant from the MWL (used as a control sample), and reference sand was assessed using Phytotoxkit F^TM^. Despite efforts to remove stones and debris from the soil to minimize impurities, the soil samples exhibited inhibitory effects for plant growth (Figure 3).

The control soil samples collected from a location distant from the MWL displayed an average root length of 31.2 mm, whereas the soil samples from the MWL exhibited an average root length of only 3.7 mm. Similarly, the reference sand samples had shorter roots compared to the control soil samples (25.3 mm for reference sand compared to 31.2 mm for the control sample). The highest toxicity was represented by all the soil samples from various levels. The samples from the −0.3 m, −1.5 m, and −6 m layers showed the highest toxicity (0 mm) and should therefore be considered toxic. Samples collected from the −10 m and −12 m layers represented high toxicity, as well (3.1–7.9 mm). The largest root growth was observed in the sample from −12 m (samples under the MWL) (15.6 mm), collected from the “bottom” layer, and this sample should be classified as moderately toxic.

A Microtox^®^ bioassay test was conducted to assess the potential effects of harmful substances extracted from the soil by water representing possible acute toxicity. The EC50 represents the results of the Microtox test, which assesses the potential acute toxicity of water extracts from samples for the bacterium *Allivibrio fischeri*. This test measures the toxicity of mobile compounds that have been extracted into the water phase. According to the results, samples collected from depths of −0.3 m, −1 m, −1.5 m, and −6 m, as well as the control sample, showed no toxicity. However, samples collected from the bottom of the municipal waste landfill (MWL) at depths of −10 m and −12 m, and samples taken from −12m below the MWL exhibited high toxicity. This result suggests that some toxic compounds, including those from the plastic structure (additives, etc.), leached from the waste and were transported by rainwater to the lower layers of the landfill.

Ostracodtoxkit^®^ is a “direct contact” ecotoxicity test in which organisms live, feed, and consistently remain in close contact with soil samples. The results revealed several main trends among the samples. Most samples exhibited significant mortality rates, exceeding 85%. This pattern was particularly evident in soil samples collected from layers at depths of −1 m, −6 m, −10 m, and −12 m, respectively. The control sample (ISO sample), on the other hand, demonstrated a mortality rate of 0.0% (significantly lower mortality percentages compared to the MWL soil samples). Soil samples collected from the layer beneath the MWL displayed mortality rates of 86.7% and 93%, respectively. Although these rates surpassed the 30% threshold, they are still considered to be highly toxic. An outlier among the samples was from the −0.3 m layer, with only 20% mortality. In conclusion, the results indicate varying degrees of toxicity and adverse effects on the organisms across different soil samples. Higher mortality rates in certain samples suggest potential environmental risks or unsuitable conditions for organism survival. The absence of mortality in the control group confirms the validity of the test.

## 3. Discussion

Plastics deposited in municipal waste landfills (MWL) are subjected to various environmental factors that influence the rate and mechanism of their degradation process. This study employed a range of analytical techniques to examine soil samples collected after undergoing PE (bio)degradation from different layers, representing varying degrees of decomposition. The primary objective was to identify environmental risks and determine factors that pose threats to human health. A distinctive feature of this research is the wide age range of the analyzed soil samples, spanning from 1.5 years up to 60 years, enabling the assessment of the long-term impacts of the degradation process. This is important because in the discussion of (bio)degradation, the initial focus was on the breakdown of the polymer chain under the influence of physical factors, such as UV, temperature, and/or biological factors, such as the activities of bacteria and fungi. However, this knowledge was not compatible with observations. According to some authors [19,20], the dominating influence occurs from the hydrolytic degradation side (considering additives make up up to 50% of PE’s structure). Additives are intensively removed from the polymer structure, causing cracks and delamination of the inner structure during this type of degradation due to water impact (flaking off successive layers of polymer). On a long-term scale, considering a sufficient amount of PE wastes (as plastic is on demand due to household use), excessive amounts of inorganic and organic additives are liberated to the environment. Considering the non-organic aspect of the study, basic parameters, such as pH value and moisture content, did not exhibit a clear increasing/decreasing trend among the samples. However, all the samples from the municipal waste landfill (MWL) showed increased pH values and decreased moisture content in contrast to the control sample. The layer beneath the MWL exhibited the lowest pH levels. These findings align with results observed in a previously conducted study [21,22], confirming the trend of the MWL as an old landfill with indications of long-term degradation.

**Figure 4:**
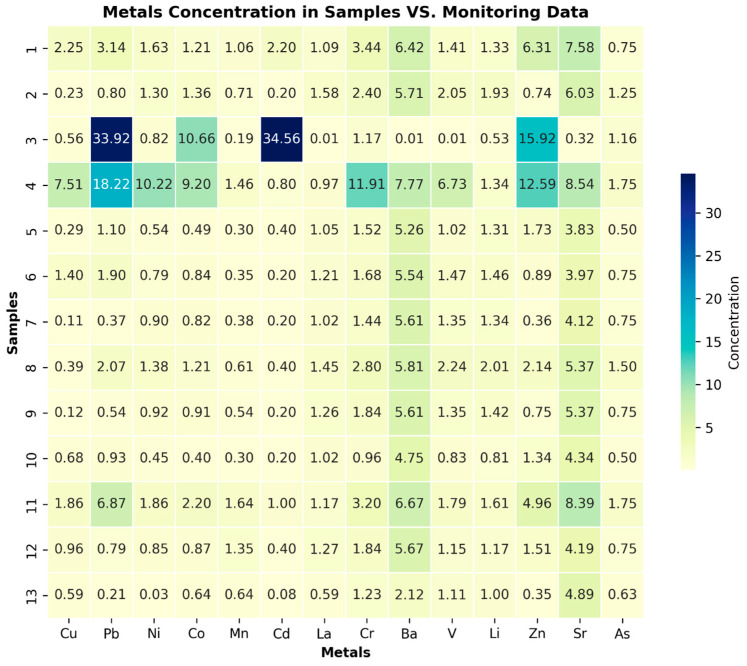
Heatmap illustrating the contrast in results compared to data obtained from the agricultural soil monitoring service. The heatmap is annotated with numbers indicating the frequency at which values in the result dataset exceed or fall below the corresponding values from the agricultural soil monitoring considered as referent data. The darker the color, the greater the contrast in concentration.

Additionally, the analysis of heavy metal revealed various tendencies and outliers in some MWL samples. High outliers were identified, particularly at a depth of −1.5 m, and the second most concentrated outliers, albeit with lower values, were found at −6 m. However, in all the samples except for the control samples, some heavy metal concentrations exceeded the established norms. Figure 4 displays the compliance of detected heavy metal levels in the MWL with data provided by the agricultural soil monitoring service in Poland. The metals’ concentrations in reference points of agricultural soil monitoring, including Cu, Pb, Ni, Co, Mn, Cd, La, Cr, Ba, V, Be, Li, Zn, Sr, and As, were compared with the results of the investigation [23]. The comparison revealed that most samples from levels above −6 m exhibited higher values, including those at −12 m. This contrast indicates that the samples from these layers are contaminated and potentially toxic.

According to the chronological scale, there was an approximate 22-year time difference between samples at depths of −1.5 m and −6 m, indicating a very slow migration of heavy metals through the layers to the bottom. Furthermore, the trend in pH levels indicates a shift towards a more acidic environment. An acidic environment significantly increases the migration and bioavailability of heavy metals, as observed in a previously conducted study [24]. In addition to this study, the bioavailability of heavy metals was assessed through extract analysis at different pH levels (5.3, 2, 1). The mobility of heavy metals in the soil mainly pertains to forms that are assimilable and bioavailable to plants and animals, allowing them to easily enter the soil solution. The potential spread of such contaminating substances in the soil environment and the threat of their infiltration into the soil solution and groundwater are particularly significant in preventing the entry of heavy metals into the food chain.

The highest availability of several metals/elements was observed at all pH values, following this order: Ca > S > Na > Mg > Zn. In some samples, elevated concentrations of Fe and Al were noted, confirming their migration. Several metals, including Zn, Fe, Al, Cu, Pb, Zn, and Ti, are used in PE production and were detected in all the samples [25]. Exceptionally high concentrations of these metals, in contrast to the MWL bottom level, were observed at depths of −0.3 m, −1 m, and −6 m. A previously conducted study [26] suggests the release of these metals during the degradation of PE plastic bags. At a depth of −0.3 m, the concentration of Ti was 325 times higher compared to the control sample (3.25 mg/L to 0.01 mg/L), indicating the liberation of this metal from the PE structure. A similar situation was observed for Zn at the same depth, with a concentration 10 times higher compared to the control sample (1159 mg/L to 109 mg/L) and three times higher at the level under the MWL (361 mg/L). Other metals, such as Pb, Fe, Cu, and Al, exhibited a similar trend, with concentrations several times higher at a depth of −12 m under the MWL, suggesting the migration of toxic metals to the bottom of the MWL. Interestingly, these layers correspond chronologically to the years 1980–1990. During the 1990s, the region underwent a transformation from a centrally planned (socialist) economy to a market-based (capitalist) economy [27]. It was during this period that a significant influx of products from developed countries in Western Europe was observed. This transformation possibly led to a significant increase in the production and demand for plastics, especially PE, since 1990, which could be a potential root cause for the deviations detected in the MWL samples. Metals such as Zn, Fe, Al, Cu, Pb, Zn, and Ti (used as UV stabilizers, antioxidants, and flame retardants) can be harmful in high concentrations. Pb is considered a toxic metal, posing a significant threat to the environment and human health. Considering previous observations and indications, a comprehensive range of toxicological tests were conducted to assess the potential toxicity of the soil collected from the MWL. The Phytotoxkit FTM test revealed significant inhibition of plant growth in the MWL soil (except for the soil sample under the MWL). Meanwhile, the sample collected under the MWL exhibited less toxicity compared to the control sample, suggesting a potential for phytotoxicity in the MWL soil and a long-term migration prospect of contaminants. In contrast, the control sample yielded even better results when compared to the ISO reference sand, possibly due to the presence of fertilizing substances.

Since plants possess numerous biochemical protection mechanisms and properties, both the Microtox^®^ bioassay and Ostracodtoxkit^®^ were employed to assess the toxic potential of the MWL soil. The Microtox^®^ test showed significant acute toxicity at a depth of soil from the −12 m level and soil under the MWL, correlating with the previously mentioned findings regarding heavy metals. Furthermore, acute toxicity was detected at levels below the MWL, implying the potential migration of toxic substances through the MWL layers. Despite the implementation of both tests, the Ostracodtoxkit^®^, being a “direct contact,” user-friendly, and highly sensitive test, exhibited consistently high mortality rates in all the samples (>30%). However, the control sample recorded less than 85%: it showed no mortality (0.0%) and is considered non-toxic, while the remaining samples are categorized as highly toxic. According to another study [28], Cd and Hg exhibited the highest toxicity effects. Although the concentration of Cd was extremely high at a depth of −1 m, the other metals did not show significant deviations when compared to the control sample. However, the sample collected under the MWL displayed a 2.5 times higher concentration of Cd compared to the control sample. Additionally, following the data from the study [29], the toxicity effects were observed in the following order: Cd > Hg > Cu > Cr > Ni ≈ Mn > Zn > Pb > Li > Fe. Most of these metals were detected in the MWL samples (even at the level under the MWL) and may possibly be liberated as additives from the PE structure. The concentration of these listed metals was significantly higher in the MWL samples than the EC50 value mentioned in the study for *H. incongruens*. This result suggests the presence of toxic/heavy metals, or the presence and migration of potentially toxic substances liberated from the PE structure due to (bio)degradation.

The analysis of ionic content (Figure 1) revealed some outliers. Particularly noteworthy trends were observed for anions, including ${SO}_{4}^{-2}$, ${Cl}^{-}$ and ${NO}_{3}^{-}$. Significant concentrations of ${SO}_{4}^{-2}$ were detected at depths of −0.3 m, −1 m, and −6 m, and declined at lower levels. A similar trend was observed for ${Cl}^{-}$, with the highest concentration at the −1 m and −6 m levels. A cationic analysis showed significant differences in ${Na}^{+}$ and ${Ca}^{2+}$ at the same levels. However, these cations are typically found in soil as nutrient compounds.

In a previous study [30], increased concentrations of ${Na}^{+}$ and ${Ca}^{2+}$ in the soil of NW Victoria were detected, which inhibited plant growth and correlated with the results of the Phytotoxkit F^TM^ test in the MWL soil investigation. Nevertheless, according to some authors [31,32], substances like calcium carbonate and talc are used as stabilizers and fillers in PE production and may be released from plastic structures during (bio)degradation. Additionally, sulfur-based substances are used as additives in PE production. The significantly increased concentration of ${SO}_{4}^{-2}$ could be linked to the liberation of additives from the PE structure. As outlined in an overview [13], antioxidants often contain sulfur compounds to protect LDPE from oxidation and degradation due to heat and oxygen exposure. Some flame retardants used in LDPE formulations may contain ${Cl}^{-}$ compounds to reduce plastic flammability. Stabilizers like calcium stearate or zinc stearate may contain ${Cl}^{-}$ or sulfur compounds to enhance thermal stability. Lubricants used in LDPE processing may contain ${Cl}^{-}$ or sulfur-based compounds to improve flow properties during manufacturing. Substances such as barium sulfate (BaSO_4_) and sodium sulfate (Na_2_SO_4_) are also used as fillers in LDPE. Long-term (bio)degradation of PE can result in the release of numerous metals (including heavy metals) and various chemical compounds, as observed in the MWL soil study. Furthermore, these elements and compounds can affect the environment, as they are typically found in natural conditions and participate in various biochemical processes. This is particularly relevant to microbiome activity. As suggested by other authors [32,33], various species of bacteria and fungi interact with plastic structures during degradation. In a previous study [34], microorganisms colonized plastic structures after 5 years of (bio)degradation and displayed increased activity, including pathogenic microorganisms. Additional evidence of increased microbiome activity could be the higher detected concentrations of ${NO}_{3}^{-}$ and ${NH}_{4}^{+}$. This may indicate nitrification processes in the soil [35]. Soil samples collected from the level below the MWL represented the second-highest concentration, with 0.61 mg/g for ${NO}_{3}^{-}$, after the samples from the −1 m level, which had a concentration of 0.72 mg/g. Furthermore, they exhibited the highest concentration of ${NH}_{4}^{+}$ at 0.22 mg/g, which was 105 times higher compared to the control sample, unlike the rest of the samples. Similar trends and suggestions are described in an overview [36], providing possible evidence for this phenomenon.

Considering the potential signs of (bio)degradation product migration from surface levels (−0.3 m) down to the bottom of the MWL (−12 m), it is worth considering the possibility of microorganisms migrating to the lower reaches of landfills. This scenario introduces an added concern for both the environment and human health, given that potentially pathogenic microorganisms could infiltrate groundwater and release toxins as part of their vital activities. This phenomenon has been explored in-depth in a review [37], highlighting the role of biofilms produced by microorganisms in enhancing their survival and toxin production.

Now, we transition the focus to the results pertaining to the detection of volatile organic compounds in both soil and air, as this forms an integral part of the research. In the initial analysis, particular attention was directed towards phthalates, widely used as plasticizers. These compounds fall under the category of EDSc (endocrine-disrupting compounds). However, phthalates constitute less than 0.02% of the PE structure, and PE cannot be considered an important source of phthalates, as noted [12].

Despite this, the highest concentrations of phthalates, including di-n-octyl phthalate (DONP), bis(2-ethylhexyl) phthalate (DEHP), and dodecyl phthalate (DDP), were found at depths of −0.3 m, −1 m, and −6 m, with the maximum observed at −0.3 m, reaching levels 105 times higher than the control sample. It is worth noting that these substances are typically used in high amounts as plasticizers in PVC (poly (vinyl chloride)). At levels below the MWL, the total concentration was 2.6 times higher compared to the control sample. Additionally, the presence of phthalates was detected in the air composition, signaling the possible migration of these substances from the soil to the air or through the layers of the MWL to the surrounding environment and groundwater, posing risks to both the environment and human health, as highlighted in previous studies [38,39].

However, when considering the results of PE (bio)degradation, the primary focus shifts to halogen-containing substances, which can account for up to 50% of the plastic structure. In all samples, bromide (${Br}^{-}$)-containing substances were detected. At levels ranging from −0.3 m to −6 m, chloro-, iodo-, and fluoro-containing substances were predominantly found. As we move deeper into the MWL, bromide-containing substances become more dominant, particularly at the bottom and under the MWL. These halogens, as discussed in paper [40], are widely used as additives in PE production, with a specific emphasis on bromide-containing substances, as mentioned in [41]. The presence of these halogens in the lower layers of the MWL suggests potential migration through the MWL’s various levels, including the MWL bottom. The heightened concentrations of these substances pose a significant risk to the environment and human health, as they can permeate through layers, including the bottom layer, and potentially reach groundwater.

An additional interesting point was observed. A large amount of polycyclic aromatic hydrocarbons (PAHs) was detected in the soil and air samples collected from MWL. The presence of such substances is a typical type of pollutant in old MWLs, as suggested by [42,43]. This observation is not directly linked to PE (bio)degradation; however, it represents an additional risk factor that requires attention. According to [43], PAHs were also detected in MWL leachates, which have the capacity to migrate through MWL layers and penetrate the environment.

Most of them possibly appeared because of fires sometimes occurring in MWLs [44] as the result of a combustion process. According to study [45], substances such as pyrene, anthracene, and fluoranthene, which form during combustion, were detected in soil and air samples, respectively. Of equal significance is the increased presence of esters, aldehydes, and alcohols in the middle levels of the MWL, indicating possibly heightened microbiological activity within those layers. In study [6], such compound classes, including esters, ketones, aldehydes, alcohols, and carboxylic acids, are associated with biomass produced by bacteria during PE (bio)degradation. It is essential to note that the presence of this biomass raises concerns about the potential migration and penetration of these substances through the MWL layers to/through the MWL bottom, with unknown toxicity prospects.

This research, made possible by the unique opportunity to collect soil samples from an old municipal waste landfill (MWL), has yielded numerous valuable insights. The primary trend that emerged was the presence of numerous outliers in the data, particularly within the middle layers of the MWL. This trend aligns with the economic transformation in Poland during the 1980s and 1990s and changes in lifestyle. Additionally, it raises concerns about the potential migration of pollutants through these layers, increasing the likelihood of these pollutants infiltrating the environment and posing risks to human health.

On one hand, we observe a rise in the quantity of PE waste, driven by the decreasing lifespan of plastics and high demand from households. On the other hand, we are confronted with the disposal of such waste in numerous older municipal waste landfills, which are not as effectively protected as modern ones. The migration of pollutants, particularly heavy metals (which exhibit slow migration), toward the deep layers and into the bottom underscores the critical importance of renovating these sites, implementing robust monitoring systems, and optimizing waste management practices to mitigate the numerous risks to the environment.

Another noteworthy pattern pertains to the activity of microorganisms. Some studies have suggested the possibility of microorganisms infiltrating the PE structure, despite the initial factor being hydrolytic degradation that leads to structural delamination and the removal of additives. Microorganisms have the capacity to create biofilms within the plastic structure, enhancing their own survival. This opens the door to the potential penetration of such microorganisms, including pathogens, into the environment through the MWL bottom, further compounding the multitude of risk factors.

## 4. Materials and Methods

### 4.1. Sample Collection

The soil and air samples were collected during the rebuilding of the municipal waste landfill (MWL) in October and November 2020. The samples were collected within two sampling campaigns at the municipal waste landfill (MWL), situated a few kilometers from Gdansk in the Pomeranian Voivodeship. Established in 1959, the landfill has received over 387,000 tons of waste, including 297,000 tons of household waste, 57,600 tons of construction waste, and 32,400 tons of industrial waste. The primary waste types deposited include mixed municipal, electronic, organic, construction waste, and materials from waste segregation. The wastes were segregated by type prior to deposition, facilitating targeted sampling of the soil and air in areas isolated from electronic or biological wastes. This segregation practice was crucial for minimizing external contamination during environmental sampling. The soil and air samples were also collected near plastic waste, enabling further studies on potential contaminant interactions. The results from these samples have been analyzed and discussed in another paper currently in publication.

**Figure 5:**
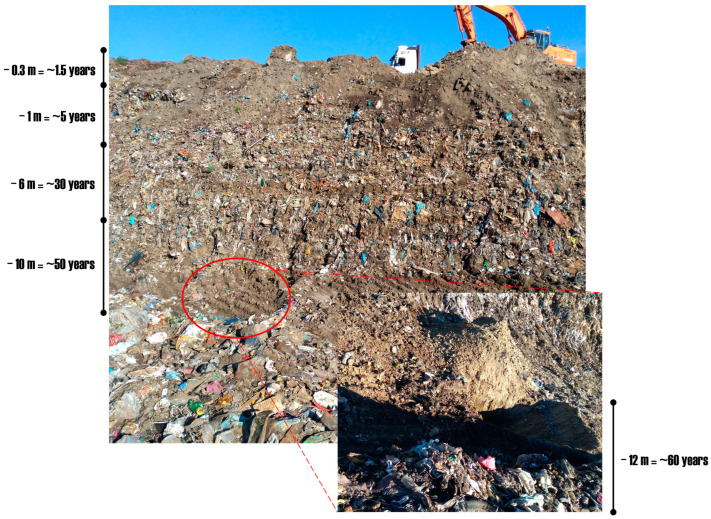
This schema illustrates the collection of soil samples from the municipal waste landfill (MWL), including the bottom layer of the pit (indicated by a red ring), which corresponds to various (bio)degradation periods.

During the sampling campaign, the first sampling on 10 October 2020 recorded an air temperature of 17 °C and a humidity level of approximately 90%. The second sampling, conducted on 14 November 2020, documented a temperature of 12 °C and humidity around 87%. In total, 13 soil samples and 5 air samples were collected. Notably, the air samples were all collected during the first sampling only, whereas each campaign involved collecting soil samples from various layers (according to the same schema as the air samples), including a control sample from a location distant from the municipal waste landfill (MWL). It is important to note that the soil samples were collected near the polyethylene (PE (PELD)) samples undergoing biodegradation described in Table S1 (such plastic samples were collected, analyzed, and described in a separate manuscript). The collection strategy focused on layers exhibiting different degrees of biodegradation, as illustrated in Figure 5.

This methodical approach to collection allowed us to successfully achieve our primary objective: the identification of environmental risks stemming from the (bio)degradation process of plastic waste over a span of approximately 60 years. Following collection, all the samples were promptly transported to the laboratory and stored at a temperature of 3 °C to prevent external contamination. All the plastic samples were covered by layers of soil, which means that anaerobic degradation dominated. The age of the samples was estimated based on the waste landfill’s layering. According to information received from the main manager, the landfill has been operational since 1960. The specific layer from which the samples were collected corresponds to a designated decade, allowing us to chronologically categorize the collected samples. This approach facilitates a unified analysis of the samples throughout the study, considering the chronological scale of waste deposition and degradation.

### 4.2. Soil Characterization

In relation to the soil where the PW (plastic waste) was deposited, the WRB soil classification system [46,47] was employed to classify the soil within the landfill and its surrounding area as Brunic Arenosols. The duration of soil involvement in (bio)degradation was determined based on the information provided by the main manager of the MWL. Additionally, the soil samples underwent further characterization, including pH value determination and moisture content assessment through a thermogravimetric approach.

Before analysis, the soil samples were purified to remove stones and other debris and subsequently ground. The pH value of each soil sample was determined: 25 mL of deionized water was mixed with 10 g of dried soil. This measurement was carried out with continuous agitation using a magnetic stir bar and a calibrated pH meter (S210, SevenCompact^TM^, Legnica, Poland) at room temperature. Within the thermogravimetric approach, 10 g of soil samples were dried at 105 °C for 48 h. The soil’s weight was measured using an analytical balance (Radwag AS160.3Y, Macclesfield, UK).

### 4.3. Anionic and Cationic Composition of Soil

The extraction of ions from the soil samples was conducted in 50 mL vials by mixing the dried soil with deionized water in a ratio of 1:10 *v*/*v*. The mixtures were shaken in a shaker incubator (Cole–Parmer Ltd., Sl 500, Vernon Hills, IL, USA) at 10 rpm for 24 h at room temperature [48]. This method of sample preparation was performed for both the anionic and cationic analyses.

Deionized water (resistance 18.2 MΩ·cm at 25 °C) from a demineralizer R5 UV (Hydrolab, Straszyn, Poland) was used for the ion extraction and preparation of all the standard mixtures and eluent. The eluent was always freshly prepared by dissolving 3.6 mmol of Na_2_CO_3_ (99.95-100.05% ACS reagent, Sigma Aldrich, Buchs, Switzerland) in one liter of deionized water. Next, the eluent was filtered through a 0.22 µm pore size membrane PTFE filter (Chemland, Stargard, Poland). The main stock solutions of anions ($F^{-}$, ${Cl}^{-}$, ${NO}_{2}^{-}$, ${Br}^{-}$, ${NO}_{3}^{-}$, ${SO}_{4}^{-2}$, ${PO}_{4}^{-3}$) and cations (${Li}^{+}$, ${Na}^{+}$, ${NH}^{4+}$, $K^{+}$, ${Mg}^{2+}$, ${Ca}^{2+}$) used for calibration were prepared from certified commercial solutions of 1000 ± 2 mg/L ion concentration (TraceCERT^®^, Supelco, Bellefonte, PA, USA). The calibration standard solutions were prepared through successive additions of the principal standard solution to deionized water, and its further dilution and was conducted by applying the customized method. The employed ion chromatography system was supplied by Shimadzu (Kyoto, Japan) with a conductometric detector (CDD-10 AVP). The system contains the following modules: controller, LC–20 ADSP pump, Rheodyne 9725i PEEK injector, and CTO-20A thermostat. The suppression of the conductivity of the eluent was performed by an XAMS anion membrane suppressor with ASUREX–A100 automatic regenerator (Diduco AB, Umeå, Sweden). Separation columns were used: Shodex SI–52 4E (PEEK) for inorganic anions and Shodex IC YS–50 for inorganic cations. The carrier material for inorganic anions containing quaternary ammonium functional groups (particle size 5 µm, column dimensions 250 mm × 4mm) was taken up. The resultant eluents were 3.6 mM Na_2_CO_3_ at a flow rate of 0.8 mL/min in the case of anions and 4 mM solution of methane sulfonic acid (99%, Sigma–Aldrich, St. Louis, MO, USA) in the case of cations with deionized water (0.05 µS/cm—Hydrolab system—Poland)). The injected sample volume was 20 µL for each sample. The column and suppressor were heated to 40 °C during analysis. The quality data from the results were obtained by calculation by applying calibration curves for each anionic ion. Considering the experiment included studying ${Ca}^{2+}$, the use of glass vials was avoided.

### 4.4. Heavy Metals Analysis

A heavy metal analysis was performed on each soil sample collected from the MWL. This study was initiated and conducted in collaboration with Bureau Veritas Commodities Canada Ltd. (Montréal-Est, QC, Canada). The used multi-acid digestion packages can dissolve most minerals. A 0.25 g split of the sample was heated in a mixture of HNO_3_, HClO_4_, and HF until fuming and then dried. The resulting residue was dissolved in HCl. The prepared extracts were then subjected to analysis using the ICP-ES/MS technique, which provides comprehensive values for most elements, such as molybdenum (Mo), copper (Cu), lead (Pb), zinc (Zn), silver (Ag), nickel (Ni), cobalt (Co), manganese (Mn), arsenic (As), uranium (U), thorium (Th), strontium (Sr), cadmium (Cd), antimony (Sb), bismuth (Bi), vanadium (V), lanthanum (La), chromium (Cr), barium (Ba), tungsten (W), zirconium (Zr), cerium (Ce), tin (Sn), yttrium (Y), niobium (Nb), tantalum (Ta), beryllium (Be), scandium (Sc), lithium (Li), rubidium (Rb), hafnium (Hf), indium (In), rhenium (Re), selenium (Se), tellurium (Te), and thallium (Tl).

The analysis of heavy metal detection facilitated the identification of metal mobility and their bioavailability in the soil environment. The sample preparation involved extract preparation from soil samples of different pH values (pH = 1, pH = 2, and pH = 5.3). This variation in pH allowed us to assess the mobility of heavy metals in soil, mainly concerning those forms that are assimilable and bioavailable to plant organisms, which can easily penetrate the soil solution. This analysis was conducted using the ICP-MS technique, which allowed for the testing of metal concentrations in soil extracts at various pH levels. Both types of analyses were conducted in ISO-certified laboratories to ensure the quality of the research and adhere to rigorous quality assurance standards.

### 4.5. Volatile and Semi-Volatile Organic Compounds

The chemical composition analysis of the soil samples was conducted using gas chromatography coupled with tandem mass spectrometry (GC–MS/MS) equipment from Shimadzu Corp., Kyoto, Japan. The decision to employ a mass spectrometer equipped with a triple quadrupole as a detector was taken to enhance the screening accuracy, reduce identification errors in the substances, and counteract matrix effects. The analysis procedure commenced with the purification of the soil samples, involving the removal of stones and other impurities. In the next stage, dichloromethane was added to the soil samples in a 1:3 ratio (3 g of soil sample + 9 mL of dichloromethane). After extraction, the soil extracts were transferred to micro-inserts (with a volume of 10 µL), firmly integrated components within a vial, and subjected to further evaporation using a stream of nitrogen.

For the analysis, 3 µL of the prepared samples were injected into the GC–MS/MS system using the spitless mode. The chromatograms derived from the soil samples were processed using LabSolutions software (version 4.45, Shimadzu Corp., Kyoto, Japan). The equipment employed for this analysis consisted of a gas chromatograph (GC-2010 PLUS, Shimadzu Corp., Japan) equipped with an autosampler (Auto Injector AOC-20ia, Shimadzu Corp., Japan) and tandem mass spectrometry (MS–TQ8040, Shimadzu Corp., Japan). The GC separation was carried out using a capillary column, specifically, Zebron^TM^ (ZB–5 MSi), measuring 30 m in length, with an inner diameter of 0.25 mm and a film thickness of 0.25 µm, supplied by Phenomenex (Torrance, CA, USA).

The inlet temperature was set to 250 °C. Helium (purity 99.999995%) used as a carrier gas was supplied by Air Products (Warsaw, Poland). The flow of the carrier gas was 1 mL min^−1^, with constant flow conditions being observed throughout. Chromatographic separation was performed in standard conditions, with the temperature program set from 40 °C to 280 °C (at 10 °C min^−1^) with the temperature held at 280 °C for 4 min. The total run time was 35 min.

The mass spectrometer was operated in full-scan mode in the range of 45–450 *m/z* with a solvent delay of 3 min. The MS conditions were the following: ion source temp., 220 °C; interface (transfer line to the tandem MS) temp., 300 °C; ionization voltage, 70 V; emission current, 150 µA. The control and operation of the chromatographic system were performed using GCMS Real Time Analysis software ver. 4.31 (Shimadzu Corp., Japan). The GC–MS data were processed with the GCMS Postrun Analysis software ver. 4.31 (Shimadzu Corp., Japan). The compound identifications were performed by using similarity searches in the National Institute of Standards and Technology MS database (NIST 11).

### 4.6. Volatile Organic Compounds’ (VOCs) Composition in Air

The VOCs’ content in the air samples collected from the MWL was tested. The air samples were collected at five different depth levels: −0.3 m, −1 m, −6 m, −10 m, and −12 m (including soil under the MWL). To avoid external contamination, the air sample collection was performed in an isolated sampling area with a domed metal barrier. The air collection was started just after the equilibrium between phases was set. The samples were adsorbed on a solid sorbent, Tenax TA prepared in quartz tubes (130 mg of Tenax TA, 1/4” × 90 mm) (Shimadzu Corp., Kyoto, Japan), according to the gas desorption method [49]. One liter of air was passed through the tubes filled with the sorbent using a hand pump. Then, the tubes were closed with metal nuts and transported to the laboratory at approx. 15 °C. The VOCs’ content was analyzed 4 h after sampling, with TD–GC–MS (thermal desorption and gas chromatography coupled with a mass spectrometer (Shimadzu Corp., Kyoto, Japan) with conditions as follows: as a carrier gas, helium (purity 99.9999%) was used and maintained at a constant flow of 1.0 mL min^−1^. The GC separation was performed on a capillary column Zebron^TM^ (ZB–5 MSi), 30 m (length): 0.25 mm (I.D.): 0.25 µm film thickness supplied by Phenomenex (Torrance, CA, USA). Within the investigation, 1-bromo-3-fluorobenzene was implemented as an internal standard for quantity analysis. The analysis aimed for an air quality comparison between the samples; thus, no internal standard was applied to a blank sample. The chromatograms were analyzed with Lab Solutions software (version 4.45, Shimadzu Corp., Kyoto, Japan).

### 4.7. Ecotoxicity Assessment

Three bioassays were performed to identify the impact of the collected soil samples on the biota using Phytotoxkit F^TM^ (MicroBioTest Inc., Gent, Belgium), Microtox^®^ bioassay (Model 500 analyzer operating with Microtox Omni Software, ver. 4.2, ModernWater Ltd., York, UK) and Ostracodtoxkit^®^.

Phytotoxkit F^TM^ is a seed germination and early root growth test that uses a higher plant of monocot (*Sorghum saccharatum*). The test was conducted according to the standard operating procedure [50] compliant with ISO 11269-1 [51]. The root length was measured after 72 h of incubation in darkness at 26 °C with ImageJ software [52]. The Microtox^®^ bioassay test is an acute toxicity bioassay based on marine, luminescent, Gram-negative bacteria *Aliivibrio fischeri*, which produce visible light as the result of their normal metabolic processes. The soil samples were mixed with deionized water (10 mL of soil with 40 mL of water (1:4 ratio)) and shaken for 18 h at 120 rpm (Cole-Parmer Ltd., Sl 500, Vernon Hills, IL, USA) at room temperature. The extracts were centrifuged (15 min; 4200 rpm; Eppendorf 5810, Hamburg, Germany), and the pH value of the supernatant was measured and adjusted if necessary to be optimal for the bacteria values (pH: 6.0–8.5) with 0.1 M HCl or 0.1 M NaOH. The toxicity of the samples was assessed according to the manufacturer’s procedure using the 81.9% screening test protocol. The concentration of the sample that produces a 50% decrease in light emission after 15 min and 30 min incubation was displayed as the EC50 values. Quality assurance and quality control were ensured by mixing control bacteria with a [ZnSO_4_ + 7H_2_O] test mixture, with 4 dilutions in 3 repetitionfor 15 min of contact.

Ostracodtoxkit^®^ is a “direct contact” ecotoxicity test in which organisms live, feed, and consistently remain in close contact with soil samples. The conducted test indicated chronic toxicity of the soil samples collected from the municipal waste landfill (MWL). The toxicity of the soil samples collected from the MWL was evaluated using the crustacean organism *Heterocypris incongruens* as the biotest organism. The test proved to be effective in assessing the quality of the environmental soil samples [53]. This test was employed to assess the ecotoxicity potential of the soil samples from the MWL, including the control soil samples and the reference samples standardized by ISO.

### 4.8. Data Analysis

The data analysis with statistical data processing were conducted using Python 3.11 [54] programming language. Moreover, several frameworks were applied for such tasks: Scikit-learn framework [55] for clusterization algorithms (k-means) implementation and validation; PlotLy [56] for data visualization; and SciPy [57] and Pingouin framework [58] for statistical tests implementation.

## 5. Conclusions

In summary, the extensive investigation of an aging municipal waste landfill (MWL) has unveiled crucial insights into (bio)degradation processes and their potential repercussions on the environment and human health. The chemical analyses have revealed the presence of liberated additives, including heavy metals and halogenated organic substances, marking a significant outcome of the research. Moreover, the migration of these pollutants has been documented, with heavy metals displaying a gradual, pH-dependent movement. Notably, the study has spotlighted an intriguing trend in concentration outliers within the middle layers of the MWL. This trend appears closely tied to the economic shifts in Poland during the 1980s and 1990s. The movement of pollutants, notably, heavy metals and additives, poses environmental threats, fueled by the mounting deposition of PE waste in older, less-protected MWLs. The ecotoxicity assessments, conducted with sensitive bioassays, unequivocally confirm the high toxicity levels prevalent throughout the MWL soil, even extending beneath it. This starkly reaffirms the trend of pollutant migration, necessitating immediate attention. Furthermore, this research underscores the potential infiltration of microorganisms, potentially including pathogens, into PE structures, where they form resilient biofilms. This phenomenon raises concerns about their potential escape into the wider environment through the MWL bottom. These findings underscore the pressing need for MWL site renovations, robust monitoring systems, and enhanced waste management practices. This is crucial to mitigate the environmental and health risks associated with pollutant migration and burgeoning microbial activity within aging MWLs. The study highlights the critical need for continued research in this domain, aiming to enhance environmental protection and safeguard public health effectively. Moreover, the evidence presented in this paper regarding the long-term negative impacts of additives released during PE (bio)degradation underscores the necessity of implementing comprehensive monitoring processes in Poland and similar regions lacking such measures. Additionally, the importance of conducting epidemiological studies is emphasized, particularly for the purpose of monitoring human health, especially among populations residing in close proximity to affected areas.

## Figures and Tables

**Table 1:** Descriptions of collected soil samples subjected to (bio)degradation impacts from differs layers.

Sample Code	Sampling Campaign *	Parameters of Soil Samples			
		Depth [m]	pH	H_2_O Content [%]	Calculated Sample Age *** [Years]
1	First	−0.3	8.08	9.8	Ca 1.5
2	Second	−0.3	7.99	12.8	Ca 1.5
3	First	−1	8.48	12.4	Ca 5
4	Second	−1	7.71	11	Ca 5
5	First	−1.5	8.27	8.2	Ca 7.5
6	First	−6	8.39	15.3	Ca 30
7	Second	−6	8.18	14.9	Ca 30
8	First	−10	7.81	12.1	Ca 50
9	Second	−12	8.44	8.4	Ca 60
10	First	−12	8.34	7.9	Ca 60
11	First	−12 (under MWL)	7.77	11	➢ 60
12	Second	−12 (under MWL)	7.75	13.0	➢ 60
13	First/Second	Control sample **	7.24	17.7	None

* The first sampling campaign (10 October 2020) and the second sampling campaign (14 November 2020); ** control sample—sample collected from distant point of MWL; *** the time a sample underwent (bio)degradation.

## Data Availability

Data are contained within the article and Supplementary Materials.

## Supplementary Materials

The following supporting information can be downloaded at: https://www.mdpi.com/article/10.3390/molecules29112499/s1, Table S1: Identification of collected plastic samples obtained with Differential Scanning Calorimetry (DSC) analysis.
