# Supplementary material for: Long-Term Polyethylene (Bio)Degradation in Landfill: Environmental and Human Health Implications from Comprehensive Analysis
Source: Molecules. 2024 May 25;29(11):2499. doi: 10.3390/molecules29112499 (PMC11173707; doi:10.3390/molecules29112499)
Supplement: Supplementary file 1 [file molecules-29-02499-s001.zip › molecules-3022027-supplementary.pdf]

Supplementary Table S1. Identification of collected plastic samples obtained with Differential Scanning Calorimetry (DSC) analysis.

Plastic sample nr.	Crystallinity degree [%]		Identification	Matching [%]
	I heating	II heating		
1	33.3	28.7	PE_LLD	80.7
2	37.8	33.2	PE_LD	96.9
3	35.8	35.6	PP	87.5
4	35.1	31.4	PE_LD	96.8
5	33.4	33.4	PE_LD	97.0
6	28.7	28.5	PE_LD	85.2
7	31.6	31.0	PE_LD	87.0
8	32.5	29.6	PE_LD	96.6
9	38.8	34.2	PE_LD	97.5
10	51.4	48.8	PE_HD	96.0
11	38.1	32.7	PE_LD	92.7
12	28.1	22.0	PE_LD	83.6
13	35.7	31.2	PE_LD	96.9
14	32.1	31.2	PE_LD	97.5
15	53.2	51.3	PE_HD	92.0
16	52.3	58.3	PE_UHMW	97.0
17	29.8	28.0	PE_LLD	83.1
18	34.8	31.8	PE_LD	94.1
19	36.0	36.0	PE_LD	96.5
20	34.3	32.5	PE_LD	97.7
21	51.8	50.4	PE_HD	96.3
